# Supplementary material for: Content-rich biological network constructed by mining PubMed abstracts
Source: BMC Bioinformatics. 2004 Oct 8;5:147. doi: 10.1186/1471-2105-5-147 (PMC528731; doi:10.1186/1471-2105-5-147)
Supplement: Additional File 5 — The original Chilibot query results of the term "long-term potentiation (LTP)" and 22 other terms, limiting the latest references analyzed to the years 1990, 1995, 2000, and 2004. [file 1471-2105-5-147-S5.bz2 › chilibotAdditionalFile5/ltp2004/html/LTP_CAMKII.html]

 


 **LTP** and **CAMKII** 
  
Found 124 abstracts in PubMed,  **30 abstracts were retrieved and analyzed**.  


---

 Search Google  |
 PDF files only 
|  EDU domain only 

---

**Interactive relationship** (e.g. stimulation, inhibition, etc)

- Mimicking inhibitory phosphorylation dramatically decreased the association of  **CaMKII**  with the PSD and blocked both  **LTP**  and learning.  Ref: 12408851 Neuron, 2002
- 3 UTR mutant mice exhibit decreases in alpha  **CaMKII**  protein in postsynaptic densities, and deficits in late phase  **LTP**  and in memory consolidation.  Ref: 12408837 Neuron, 2002
- Conversely, blockade of  **CaMKII**  activity prevented the synaptic enhancement, the growth of filopodia and formation of new spines triggered by  **LTP**  induction, and a short anoxia hypoglycemia.  Ref: 14627649 J Neurosci, 2003
- However, inhibition of  **CaMKII**  together with inhibition of either PKA or PKC fully blocked the induction of  **LTP** .  Ref: 14529720 Neuropharmacology, 2003
- These experiments reveal, unexpectedly, the existence of two parallel kinase pathways, one involving  **CaMKII**  and the other PKA and PKC, either of which can fully support the induction of  **LTP** , at this stage of development.  Ref: 14529720 Neuropharmacology, 2003
- These results suggest that activation of  **CaMKII** , PKA and PKC may be crucial for the induction and the early phase but not for the late phase maintenance of the spinal  **LTP** .  Ref: 14586032 J NeurophysiolJ Neurophysiol, 2003
- we found that high concentrations of D 890 can directly inhibit calmodulin protein kinase II  **CaMKII** , an enzyme required for  **LTP**  induction.  Ref: 12440581 Hippocampus, 2002
- We show that Ras relays the NMDA R and  **CaMKII**  signaling that drives synaptic delivery of AMPA Rs during long term potentiation  [ **LTP** ] .  Ref: 12202034 Cell, 2002
- Synaptic accumulation of AMPA receptors containing the glutamate receptor 1 subunit can be driven by calcium calmodulin dependent protein kinase II  [ **CAMKII** ]  activity or long term potentiation  [ **LTP** ]  and requires an interaction between GluR1 and a type I PDZ domain containing protein.  Ref: 12097490 J Neurosci, 2002
- The data suggest that the dentate gyrus of chronically stressed rats may have developed a compensatory mechanism whereby calcineurin levels are reduced to maintain normal P  **CaMKII**  levels, which may be responsible for the normal early  **LTP**  of the dentate gyrus of chronically stressed rats.The results of this work will increase understanding of why certain brain regions are more resistant to deleterious effects of conditions that deteriorate learning and memory.  Ref: 12654339 Neuroscience, 2003
- Considerable evidences have shown that calcium calmodulin dependent protein kinase II  [ **CAMKII** ]   **CaMKII** , protein kinase A PKA and protein kinase C PKC are important for  **LTP**  in hippocampus.  Ref: 14586032 J NeurophysiolJ Neurophysiol, 2003
- The results support a model in which locally stored  **camkII**  messenger ribonucleic acid is rapidly transported to dendritic spines and translated during long term potentiation  [ **LTP** ]  in behaving rats.  Ref: 12823475 Eur J Neurosci, 2003
- Our data demonstrate that blocking inhibitory phosphorylation increases  **CaMKII**  in the postsynaptic density PSD, lowers the threshold for hippocampal long term potentiation  [ **LTP** ]   **LTP** , and results in hippocampal dependent learning that seems more rigid and less fine tuned.  Ref: 12408851 Neuron, 2002

**Parallel relationship** (e.g. studied together, co-existance, homology, etc.)

- In present work the roles of these three protein kinases in the induction and maintenance of  **LTP**  of C fiber evoked field potentials were evaluated by application of specific inhibitors of  **CaMKII**  KN 93 and AIP, PKA Rp CPT cAMPS and PKC chelerythrine and Go 6983 at the recording segments before and after  **LTP**  induction in Urethane anesthetized Sprague Dawley rats.  Ref: 14586032 J NeurophysiolJ Neurophysiol, 2002
- We report here that, unlike  **LTP**  in the more mature CA1 region of the hippocampus,  **LTP**  in neonatal rodent hippocampus < ; 9 postnatal days, < ; P9 requires cyclic AMP dependent protein kinase A PKA but not calcium calmodulin dependent protein kinase II  **CaMKII** .  Ref: 12469130 Nat Neurosci, 2003
- Bursts of high frequency stimulation trigger rapid delivery of pre existing alpha  **CaMKII**  mRNA to synapses a mechanism in dendritic protein synthesis during long term potentiation  [ **LTP** ]  in adult awake rats.  Ref: 12823475 Eur J Neurosci, 2003
- These results demonstrate that in the hippocampus,  **CaMKII**  activation facilitates the induction of low frequency  **LTP** , but at high levels of expression, compensatory mechanisms act to inhibit the induction of this form of  **LTP** .  Ref: 12097524 J Neurosci, 2002
- Mice expressing low levels of a  **CaMKII**  Asp286 transgene have facilitated low frequency 5 Hz induced long term potentiation  [ **LTP** ]   **LTP** .  Ref: 12097524 J Neurosci, 2002
- Molecular genetics experiments using gene targeting and transgenic technology demonstrated the importance of alpha calcium calmodulin dependent protein kinase II  [ **CAMKII** ]  alphaCaMKII in long term potentiation  [ **LTP** ]   **LTP**  and memory.  Ref: 12231258 Brain Res, 2002
- Here we explored possible  **camkII**  trafficking in the context of long term potentiation  [ **LTP** ]  in the dentate gyrus of awake, adult rats.  Ref: 12823475 Eur J Neurosci, 2003
- Because the experimentally observed inactivation of  **CaMKII**  by PP1 accounts for this latter characteristic, we aimed at verifying whether a realistic model using reported parameters values can simulate the induction of either  **LTP**  or LTD, depending on the time and amplitude characteristics of the calcium signal.  Ref: 12823459 Eur J Neurosci< MedlineTA>Eur J Neurosci, 2003
- The Roles of  **CaMKII** , PKA and PKC in the Induction and Maintenance of  **LTP**  of C Fiber Evoked Field Potentials in Rat Spinal Dorsal Horn.  Ref: 14586032 J NeurophysiolJ Neurophysiol, 2003
- This  **CaMKII**  dependent long term potentiation  [ **LTP** ]  is important for hippocampal learning and memory.  Ref: 12928489 Proc Natl Acad Sci U S A, 2003
- Surprisingly, given the large body of evidence supporting a role for calcium calmodulin dependent protein kinase II  [ **CAMKII** ]   **CaMKII**  in  **LTP** , inhibition of this enzyme did not affect the induction of  **LTP**  at this age.  Ref: 14529720 Neuropharmacology, 2003
- Activation of  **CaMKII**  and IV and mitogen activated protein kinase MAPK was observed during long term potentiation  [ **LTP** ]   **LTP**  induction in the CA1 area of hippocampus.  Ref: 12491766 Nippon Yakurigaku Zasshi, 2002
- The switch between the calcium dependence of LTS induction to the calcium independence of LTS maintenance is consistent with  **CaMKII**  autophosphorylation, similar to proposed mechanisms of hippocampal long term potentiation  [ **LTP** ] .  Ref: 12388588 J Neurosci, 2002
- The results suggest that  **CaMKII**  is involved in  **LTP**  induction, while CaMKIV and MAPK are rather involved in  **LTP**  maintenance.  Ref: 12491766 Nippon Yakurigaku Zasshi, 2002
